# Chitinase 3-like protein 1: a diagnostic biomarker for early liver fibrosis in autoimmune liver diseases

**DOI:** 10.3389/fimmu.2025.1504066

**Published:** 2025-04-25

**Authors:** Shafei Liu, Conggao Peng, Shijia Xia, Chaonan Li, Xiahong Dai, Xingyu Liu, Meng Zhang, Xiaoping Li, Lingling Tang

**Affiliations:** ^1^ School of Medicine, Zhejiang Chinese Medical University, Hangzhou, China; ^2^ Department of Infectious Diseases, Key Laboratory of Artificial Organs and Computational Medicine of Zhejiang Province, Shulan(Hangzhou)Hospital, Hangzhou, China; ^3^ Shulan International Medical College, Zhejiang Shuren University, Hangzhou, China; ^4^ Collaborative Innovation Center for Diagnosis and Treatment of Infectious Diseases, State Key Laboratory for Diagnosis and Treatment of Infectious Diseases, the First Hospital of Zhejiang University School of Medicine, Hangzhou, China; ^5^ School of Medicine, Affiliated Hospital of Hangzhou Normal University, Hangzhou, China; ^6^ Key Laboratory of Artificial Organs and Computational Medicine in Zhejiang Province, Shulan International Medical College, Zhejiang Shuren University, Hangzhou, China

**Keywords:** chitinase 3 like protein 1, liver fibrosis, autoimmune liver diseases, chronic HBV, fibroscan

## Abstract

**Background and Aims:**

Chitinase 3-like protein 1 (CHI3L1) is a marker of liver fibrosis produced mainly by hepatic macrophages. However, few studies have assessed the relationship between CHI3L1 and liver fibrosis in autoimmune liver diseases (AILDs). We aimed to explore the diagnostic value of CHI3L1 for liver fibrosis in AILDs and to compare its application differences between AILDs and chronic hepatitis B (CHB) patients.

**Methods:**

The fibrotic group was defined as liver stiffness measurement (LSM) > 9.70kPa. Serum CHI3L1 levels were measured by ELISA in 78 AILDs patients, 65 chronic hepatitis B patients. The diagnostic accuracy was evaluated by the area under the receiver operating characteristic curve (AUROC).

**Results:**

Serum CHI3L1 levels in AILDs patients were positively correlated with LSM (r=0.750, *p <*0.001). The AUROC for serum CHI3L1 in identifying significant liver fibrosis was 0.939 (95% CI: 0.891 - 0.988), which was higher than that of other non - invasive fibrosis scores (APRI, FIB - 4, GPR, AAR, NLP, and PLR). At the optimal cutoff value of 86.84 ng/mL, the sensitivity and specificity were 92.9% and 83.3%, respectively. Furthermore, in patients with no significant difference in LSM, serum CHI3L1 levels were higher in the autoimmune liver disease group than in the CHB group.

**Conclusion:**

Serum CHI3L1 is an effective non-invasive indicator for assessing liver fibrosis in AILDs patients and may vary in different etiologies.

## 1 Introduction

Autoimmune liver diseases (AILDs) are a spectrum of autoimmune disorders affecting the liver and biliary system, including autoimmune hepatitis (AIH), primary biliary cholangitis (PBC), primary sclerosing cholangitis (PSC), and IgG4-related cholangitis (IgG4-SC) (1). Approximately 10% of AIH or PBC patients present with an overlap of PBC/AIH, known as the PBC-AIH overlap syndrome (OS) (2). The global incidence of AILDs has been on the rise in recent years, leading to an increasing disease burden (3). Similar to other liver diseases, the progression of AILDs can be categorized into stages of hepatitis, liver fibrosis, and cirrhosis, with liver fibrosis serving as a significant predictor of disease progression and clinical outcomes in AILDs. Regular follow-up assessments are essential for guiding management and treatment strategies for patients with AILDs.

Liver biopsy is currently recognized as the gold standard for diagnosing liver diseases and evaluating the extent of fibrosis. However, it is not without drawbacks. Its invasive nature, the risk of complications, and the potential for sampling errors significantly limit its application. These limitations prevent it from being widely used in the assessment and dynamic monitoring of liver fibrosis on a routine basis (4, 5). Among non-invasive testing (NITs) methods, transient elastography is widely used and validated with good repeatability and high accuracy for diagnosing cirrhosis, making it the gold standard for non-invasive scoring. However, it requires specialized equipment and is susceptible to factors such as obesity, ascites, and operator experience, thereby reducing its overall applicability compared to serum biomarkers (6).

Several non-invasive serum markers and scores demonstrate potential for diagnosing liver fibrosis, including serum chitinase 3-like protein 1(CHI3L1), fibrosis index based on four factors (FIB-4), aspartate aminotransferase-to-platelet ratio index (APRI), aspartate aminotransferase-to-alanine aminotransferase ratio (AAR), neutrophil-to-lymphocyte ratio (NLR), the platelet-to-lymphocyte ratio (PLR), and gamma glutamyl transferase-to-platelet ratio (GPR) (7). In clinical practice, these markers offer advantages in terms of simplicity, speed, and reproducibility.

Serum CHI3L1 is a secretory protein primarily synthesized by hepatic macrophages (8), also known as YKL-40 due to the presence of three N-terminal amino acid residues in its secreted form—tyrosine (Y), lysine (K), and leucine (L) (9). Numerous studies have demonstrated the efficacy of CHI3L1 in evaluating and dynamically monitoring the extent of liver fibrosis, with current clinical applications (10, 11). Our team’s previous research has indicated that serum CHI3L1 levels increase with the progression of liver fibrosis in chronic hepatitis B (CHB) patients and outperform Hyaluronic acid (HA), type III procollagen III (PC-III), type IV collagen IV (IV-C), and laminin (LN) in diagnosing liver fibrosis (12). Limited research has investigated the role of CHI3L1 in diagnosing liver fibrosis in autoimmune liver diseases patients.

Therefore, this study aims to investigate the correlation between CHI3L1 and liver fibrosis in AILDs, compare serum CHI3L1 with other liver fibrosis assessment methods to explore its clinical application value as a non-invasive diagnostic biomarker for liver fibrosis. And by comparing the levels of serum CHI3L1 in AILDs and CHB patients with similar degrees of liver fibrosis, we investigate the patterns of changes in serum CHI3L1 in chronic liver diseases caused by different etiologies.

## 2 Materials and methods

### 2.1 Study population

We conducted an observational, retrospective, single-center study in Shulan (Hangzhou) Hospital between October 4, 2020, and July 31, 2024. The inclusion criteria were patients with AILDs who underwent serum CHI3L1 testing and Fibroscan examination. Diagnosis of AIH was based on the simplified diagnostic criteria of the International Autoimmune Hepatitis Group (13), while PBC diagnosis followed the 2017 European Association for the Study of the Liver (EASL) Clinical Practice Guidelines (14). The diagnosis of PBC-AIH OS was in accordance with relevant consensus (15). Concurrently, a control group consisting of patients with CHB was also included, diagnosed according to the 2015 Asian Pacific Association for the Study of the Liver (APASL) guidelines (16). Exclusion criteria included: (a) Patients with liver diseases of non-single etiology, such as those with alcoholic liver disease, metabolic dysfunction-associated steatotic liver disease (MASLD), and drug-induced hepatitis (17); (b) Patients with liver cancer or other malignancies; (c) Patients with diabetes, coronary artery disease, asthma, arthritis, sepsis and severe heart, lung, kidney dysfunction, or other severe primary diseases; (d) Patients with ascites or Body Mass Index (BMI)>30 kg/m²; (e) Patients under the age of 18, pregnant or lactating women, or individuals with severe consciousness disorders or mental abnormalities who could not cooperate; (f) Patients with severely missing clinical data. Finally, this study included a total of 78 AILDs patients, 65 CHB patients. The study protocol was approved by Research Ethics Committee of Shulan (Hangzhou) Hospital (Approval ID: KY2024093). According to relevant regulations, written informed consent from participants was not required. All methods were conducted in accordance with relevant guidelines and regulations.

### 2.2 Clinical assessment and laboratory tests

All patients underwent standardized medical history, laboratory assessment, anthropometric measurements, and physical examinations. Biochemical tests included measurements of neutrophil, lymphocyte, platelet, prothrombin time, albumin, aspartate aminotransferase (AST), alanine aminotransferase (ALT), gamma glutamyl transferase (GGT), alkaline phosphatase (ALP), total bilirubin. And derived parameters FIB-4, APRI, AAR, NLR, PLR, GPR according to the following formulas: FIB‐4 = age×AST(IU/L)/(platelet (10^9^./L)×ALT(IU/L)½); APRI = [(AST/ULN)× 100]/platelet (10^9^./L); AAR = AST(IU/L)/ALT(IU/L); NLR = neutrophil(10^9^./L)/lymphocyte(10^9^./L); PLR = platelet (10^9^./L)/lymphocyte(10^9^./L); GPR = [(GGT/ULN)× 100]/platelet (10^9^./L). The upper limit of normal (ULN) for AST and GGT were 40 IU/L and 45 IU/L, respectively. Imaging examinations collected included liver ultrasound, liver CT, and liver MR, among others. Liver pathological diagnosis and fibrosis staging results were also collected.

### 2.3 Definition of liver fibrosis

We selected the FibroScan 502 model liver function shear wave quantification ultrasound diagnostic instrument and the accompanying medium-sized probe (3.5 MHz) from EchoSens Company for testing. Liver stiffness measurement (LSM) was measured continuously at the 7th, 8th, and 9th intercostal spaces from the right mid-clavicular line to the mid-axillary line, with data recorded from 10 measurements and the final result calculated as the median. Measurements were deemed inadequate if they deviated by more than one-third of the median. All tests were conducted by a consistent group of attending physicians with over 5 years of experience. Fibroscan was also included controlled attenuation parameter (CAP) measurement, which could help us to assess liver steatosis. In accordance with the FibroScan 502 user manual, the degree of liver fibrosis was evaluated using LSM values, with a cutoff value of 9.70 kPa established to differentiate between non-significant and significant fibrosis (18–20).

### 2.4 CHI3L1

The serum CHI3L1 levels were detected by enzyme-linked immunosorbent assay (ELISA). Reagents comes from Hangzhou Pwang Biotechnology Co., Ltd

### 2.5 Statistical methods

Continuous variables with a normal distribution were presented as the mean ± standard deviation (SD), while those not normally distributed were expressed as the median with interquartile range (IQR). Intergroup comparisons were conducted using the Kruskal-Wallis test, one-way ANOVA, Mann-Whitney U test or Student’s *t*-test for continuous variables and the *χ*2 test for categorical variables. Receiver operating characteristic (ROC) curves was performed to evaluate the predictive accuracy of CHI3L1 and different NITs. The area under the receiver operating characteristic curve (AUROC) and 95% confidential interval (CI) of AUROC were calculated. The optimal cut-off value was selected based on the best sensitivity and specificity in the AUROC analysis (Youden’s index). Values of *p <*0.05 was considered to be statistically significant. The data were analyzed using SPSS version 25.0 (SPSS Inc., New York, NY, USA) and GraphPad Prism version 8.0 (GraphPad Software Inc., California, CA, USA).

## 3 Results

### 3.1 General characteristics of the cohort

In this retrospective study, a total of 144 patients with AILDs and CHB were included, all of whom underwent Fibroscan and serum CHI3L1 testing. Among the 78 AILDs patients, 26 (33.33%) had AIH, 30 (38.46%) had PBC, and 22 (28.21%) had the PBC-AIH OS. The mean age of the patients was 56.35 ± 10.12 years old, and the median BMI was 22.64 (IQR 21.33-24.86) kg/m². The proportion of female patients was significantly higher than that of male patients. When compared with CHB patients, there were no statistically significant differences in Child-Pugh Score, BMI, LSM, neutrophil, lymphocyte, platelet, prothrombin time, ALT, total bilirubin, FIB-4, APRI and NLR between AILDs patients and CHB patients (*p >*0.05). However, there were statistically significant differences in albumin, AST, GGT, ALP, CHI3L1, AAR, PLR and GPR (*p <*0.05). For additional information on participants, please refer to **Table 1**.

**Table 1:** Baseline characteristics of AILDs patients and control groups.

Factors	AILDs patients (N=78)	CHB patients (N=65)	*t/z*	*p*-value
Age (years)	56.35 ± 10.12	47.22 ± 10.54	5.268	<0.001
BMI (Kg/m²)	22.64 (21.33-24.86)	23.46 (21.47-27.47)	1.837	0.066
Sex			57.201	<0.001
Male (%)	11 (14.10%)	50 (76.92%)		
Female (%)	67 (85.90%)	15 (23.08%)		
Child-Pugh Score			0.242	0.886
A (%)	63 (80.77%)	54 (83.08%)		
B (%)	13 (16.67%)	10 (15.38%)		
C (%)	2 (2.56%)	1 (1.54%)		
LSM (KPa)	11.10 (6.88,20.40)	11.10 (6.85-23.30)	0.201	0.841
Neutrophil (×10^9^./L)	2.70 (1.88-3.73)	2.70 (2.15-3.40)	0.049	0.961
Lymphocyte (×10^9^./L)	1.40 (0.78-1.83)	1.40 (0.80-1.80)	0.102	0.919
Platelet (×10^9^./L)	135.00 (82.75-214.50)	129.00 (66.50-187.50)	1.316	0.188
Prothrombin time (s)	11.85 (10.88-13.20)	12.20 (11.40-13.70)	1.714	0.087
Albumin (g/L)	40.50 (35.75-43.83)	42.90 (38.60-45.70)	2.037	0.042
ALT (IU/L)	31.50 (18.00-50.00)	27.00 (19.00-35.00)	1.292	0.196
AST (IU/L)	36.50 (27.00-59.00)	27.00 (23.00-39.50)	3.341	0.001
GGT (IU/L)	58.50 (28.00-172.50)	27.00 (15.00-56.50)	4.639	<0.001
ALP (IU/L)	105.00 (87.50-161.00)	79.00 (69.00-111.50)	4.215	<0.001
Total bilirubin (μmol/L)	15.00 (11.00-22.00)	16.00 (11.50-23.50)	0.546	0.585
CHI3L1 (ng/mL)	107.25 (59.64-271.85)	86.90 (51.91-136.11)	2.392	0.017
FIB-4	3.38 (1.59-6.09)	2.10 (1.14-5.61)	1.687	0.092
APRI	0.94 (0.42-1.70)	0.71 (0.35-1.65)	0.762	0.446
AAR	1.33 (0.94-1.86)	1.16 (0.80-1.50)	2.038	0.042
NLR	1.94 (1.40-3.12)	2.13 (1.65-3.00)	0.560	0.576
PLR	112.97 (74.82-148.68)	93.70 (70.00-118.13)	2.139	0.032
GPR	1.25 (0.46-2.73)	0.59 (0.27-1.44)	3.292	0.001

AILDs, Autoimmune liver diseases; CHB, Chronic hepatitis B; BMI, Body Mass Index; LSM, Liver stiffness measurement; ALT, Alanine aminotransferase; AST, Aspartate aminotransferase; GGT, Gamma glutamyl transferase; ALP, Alkaline phosphatase; CHI3L1, Chitinase 3-like protein 1; FIB-4, Fibrosis index based on four factors; APRI, Aspartate aminotransferase-to-platelet ratio index; AAR, Aspartate aminotransferase-to-alanine aminotransferase ratio; NLR, Neutrophil-to-lymphocyte ratio; PLR, Platelet-to-lymphocyte ratio; GPR, Gamma glutamyl transferase-to-platelet ratio.

### 3.2 Correlation between serum CHI3L1 levels and liver fibrosis

**Figure 1:**
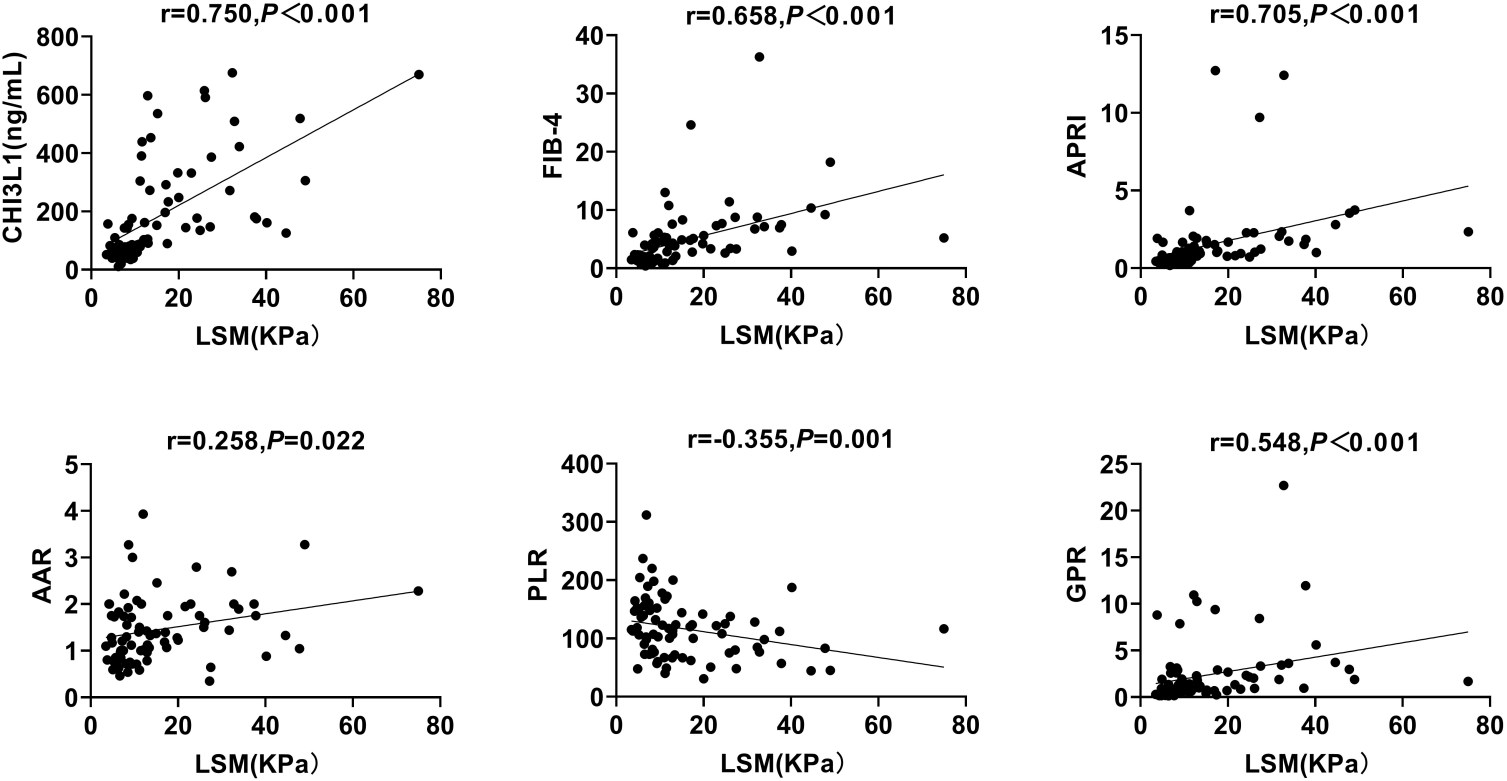
Correlation between serum CHI3L1 levels and non-invasive liver fibrosis scores with LSM in AILDS patients. Spearman’s rank correlation coefficient was used for correlation analysis. Solid line: linear growth trend; R: correlation coefficient. CHI3L1, Chitinase 3-like protein 1; LSM, Liver stiffness measurement; FIB-4, Fibrosis index based on four factors; APRI, Aspartate aminotransferase-to-platelet ratio index; AAR, Aspartate aminotransferase-to-alanine aminotransferase ratio; PLR, Platelet-to-lymphocyte ratio; GPR, Gamma glutamyl transferase-to-platelet ratio.

To evaluate the potential of CHI3L1 as a biomarker for hepatic fibrosis in patients with autoimmune hepatitis, we measured liver stiffness values (LSM), serum CHI3L1 levels, as well as other commonly used hepatic fibrosis scores (FIB-4, APRI, AAR, NLR, PLR, GPR). Through Spearman’s correlation coefficient analysis, it was found that serum CHI3L1 was significantly positively correlated with LSM (r=0.750, *p <*0.01), and the correlation was higher than that of FIB-4 (r=0.658, *p <*0.001), APRI (r=0.705, *p <*0.001), AAR (r=0.258, *p* =0.647), and GPR (r=0.548, *p <*0.001). PLR showed a negative correlation (r=-0.355, *p* =0.001) (**Figure 1**). However, no statistically significant correlation was observed between NLR and LSM (r=0.053, *p* =0.647).

### 3.3 Correlation between serum CHI3L1 levels and liver inflammation

**Figure 2:**
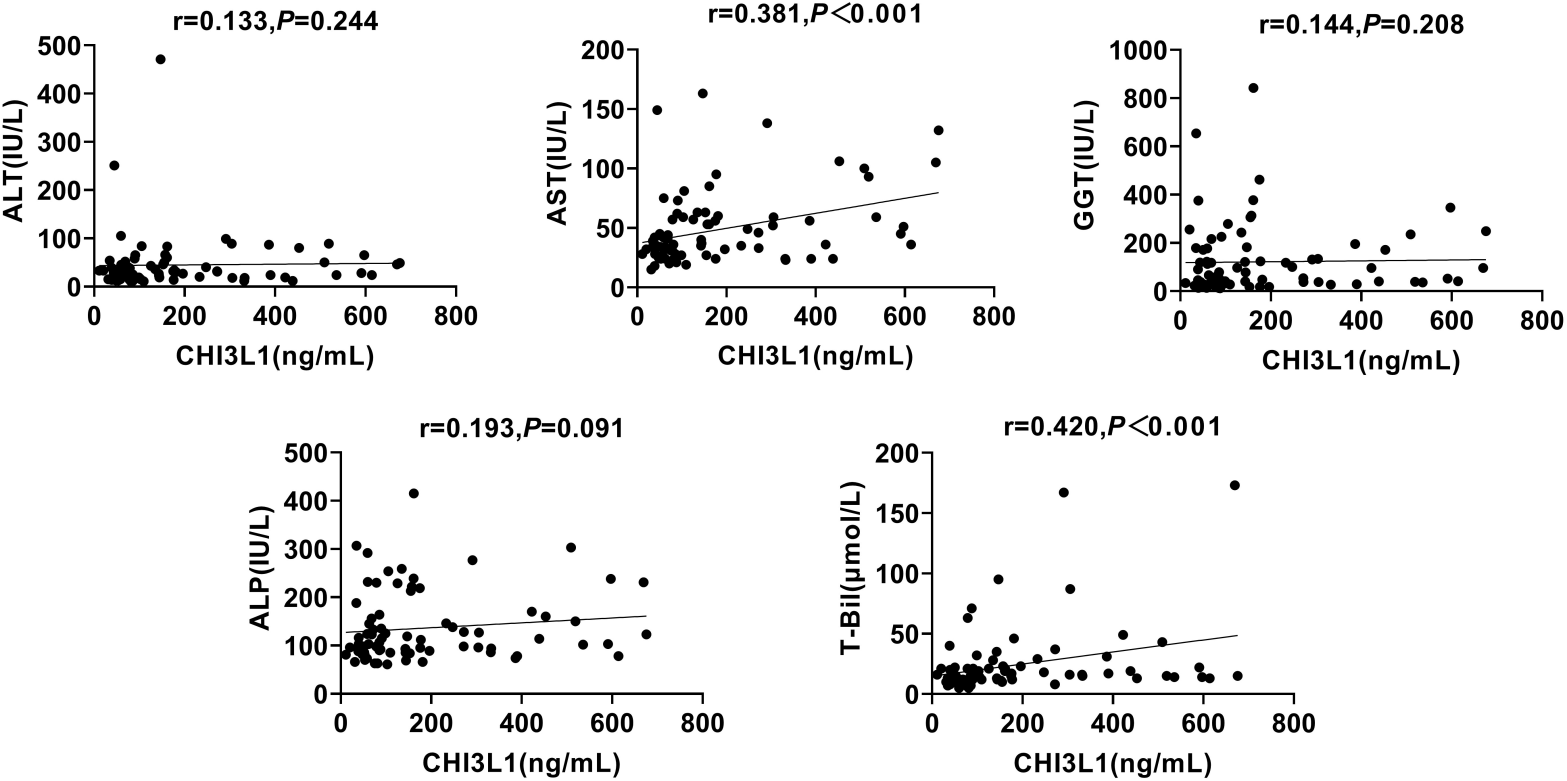
Correlation between serum CHI3L1 levels and liver inflammation markers in AILDS patients. Spearman’s rank correlation coefficient was used for correlation analysis. Solid line, linear growth trend; R, correlation coefficient. CHI3L1, Chitinase 3-like protein 1; ALT, Alanine aminotransferase; AST, Aspartate aminotransferase; GGT, Gamma glutamyl transferase; ALP, Alkaline phosphatase; T-Bil, Total bilirubin.

To investigate whether CHI3L1 is associated with liver inflammation markers, such as ALT, AST, GGT, ALP, and T-Bil, and to determine whether this biomarker is confounded by any of these factors, we conducted a Spearman’s correlation coefficient analysis. The results showed that serum CHI3L1 did not exhibit a statistically significant correlation with ALT, GGT, or ALP (*p >*0.05). However, it was significantly correlated with AST and T-Bil (r=0.381, *p <*0.001; r=0.420, *p <*0.001) (**Figure 2**).

### 3.4 Diagnostic value of serum CHI3L1 for liver fibrosis in AILDs patients

**Figure 3:**
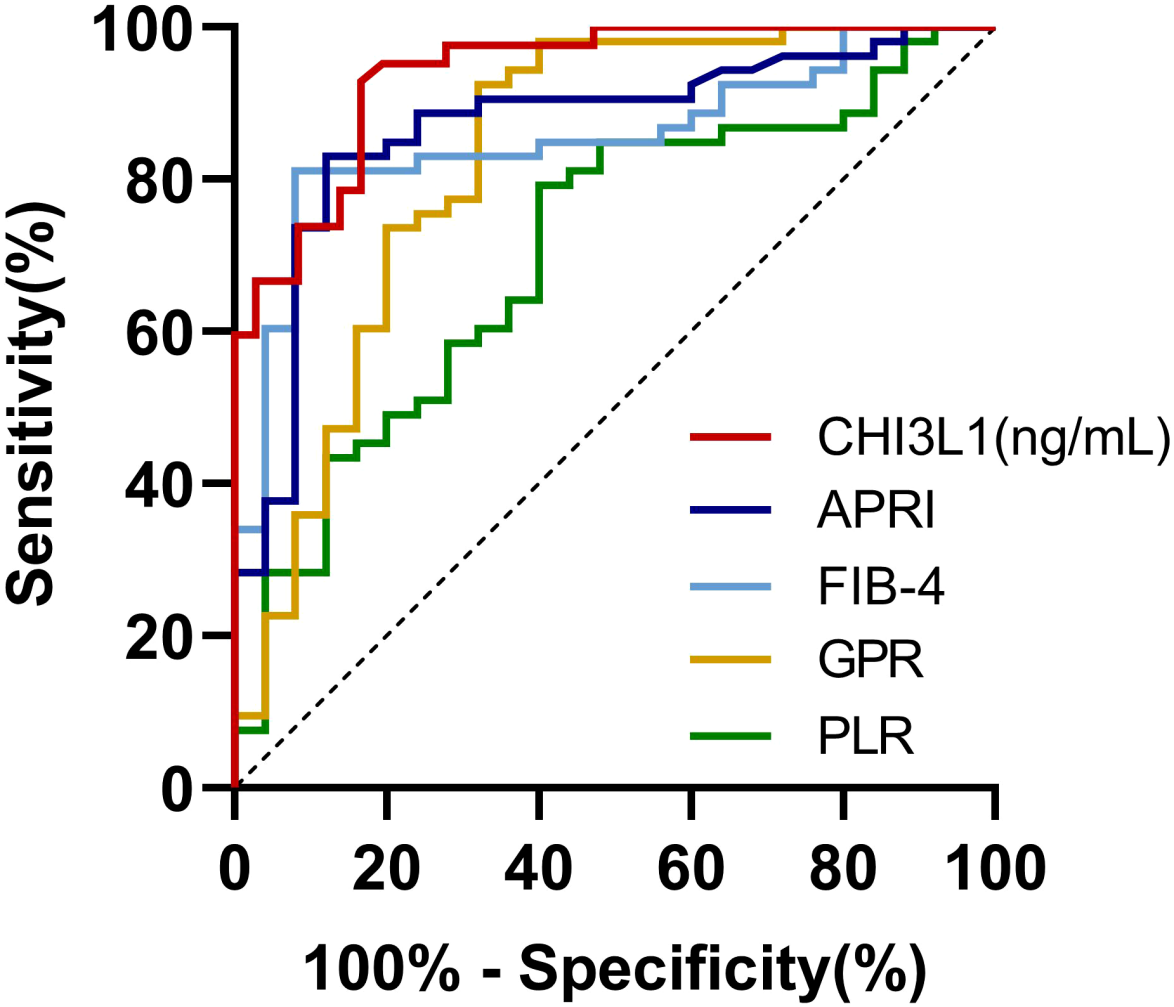
AUROCs for the ability of non-invasive testing to discriminate significant liver fibrosis in AILDs patients. AUROC, The area under the receiver operating characteristic curve; CHI3L1, Chitinase-3-like protein 1; FIB-4, Fibrosis index based on four factors; APRI, Aspartate aminotransferase-to-platelet ratio index; AAR, Aspartate aminotransferase-to-alanine aminotransferase ratio; NLR, Neutrophil-to-lymphocyte ratio; PLR, Platelet-to-lymphocyte ratio; GPR, Gamma glutamyl transferase-to-platelet ratio.

All cases were stratified into no significant liver fibrosis group and significant liver fibrosis group based on a cut-off of 9.70 kPa to determine its ability to diagnose fibrosis staging. The results demonstrated that serum CHI3L1 had the highest AUROC of 0.939 (95% CI 0.891-0.988, *p <*0.001) for the diagnosis of significant liver fibrosis, outperforming other non-invasive fibrosis scoring indicators. Additionally, the optimal cutoff value for serum CHI3L1 was determined to be 86.840 ng/mL, which is higher than the currently recognized threshold of 79 ng/mL. At this cutoff, the sensitivity was 92.9% and the specificity was 83.3% (**Table 2**; **Figure 3**).

**Table 2:** Diagnostic performances of CHI3L1, FIB-4, APRI, AAR, NLR and PLR for liver fibrosis in patients with AILDs.

Factors	Cut-off value	Sensitivity (%)	Specificity (%)	Youden’s index	PPV (%)	NPV (%)	AUROC	95%CI	*p*-value
CHI3L1(ng/mL)	86.840	92.9	83.3	0.762	86.6	91.0	0.939	0.891-0.988	<0.001
APRI	0.696	92.9	77.8	0.707	83.0	90.4	0.880	0.803-0.958	<0.001
FIB-4	2.470	88.1	77.8	0.659	82.2	84.9	0.854	0.769-0.939	<0.001
GPR	0.521	92.5	52.8	0.480	69.6	85.8	0.735	0.621-0.849	<0.001
AAR	1.012	81.0	44.4	0.254	63.0	66.7	0.618	0.492-0.745	0.074
NLR	6.834	11.9	100.0	0.119	100.0	49.3	0.517	0.388-0.646	0.798
PLR	130.072	19.0	47.2	0.073	29.6	33.3	0.332	0.211-0.453	0.011

AILDs, Autoimmune liver diseases; CHI3L1, Chitinase 3-like protein 1; APRI, Aspartate aminotransferase-to-platelet ratio index; FIB-4, Fibrosis index based on four factors; GPR, Gamma glutamyl transferase-to-platelet ratio; AAR, Aspartate aminotransferase-to-alanine aminotransferase ratio; NLR, Neutrophil-to-lymphocyte ratio; PLR, Platelet-to-lymphocyte ratio; PPV, Positive predictive value; NPV, Negative predictive value; AUROC, The area under the receiver operating characteristic curve; *CI*, Confidence interval.

### 3.5 Comparison of serum CHI3L1 levels in AILDs and CHB patients in the liver fibrosis group

We further compared patients with AILDs and CHB in the significant liver fibrosis group. The results showed that there was no statistically significant difference in LSM between two groups (*p >*0.05). In order to explore the potential differences in serum CHI3L1 levels among patients with liver fibrosis of different etiologies, we compared the CHI3L1 levels between patients with autoimmune liver diseases and CHB patients. The results revealed that the serum CHI3L1 levels were significantly higher in patients with autoimmune liver diseases than in the CHB patient group, with median values of 240.27 ng/mL (IQR 132.62 - 426.60) and 118.38 ng/mL (IQR 69.03-168.43), respectively; *p*<0.001. However, it was also observed that there were differences in liver inflammation markers, including AST, GGT, and ALP, between the two groups (**Table 3**).

**Table 3:** Comparison of AILDs and CHB in the significant fibrosis group.

Factors	AILDs(N=42)	CHB(N=37)	*t/z*	*p*-value
Age(years)	59.38 ± 9.21	48.81 ± 10.92	4.668	<0.001
BMI(Kg/m²)	23.25 ± 2.86	24.29 ± 3.39	1.481	0.143
LSM(KPa)	18.70(12.89-31.85)	18.95(12.23-38.58)	0.314	0.753
CHI3L1(ng/mL)	240.27(132.62-426.60)	118.38(69.03-168.43)	4.146	<0.001
FIB-4	5.23(3.34-8.44)	4.55(2.14-7.26)	1.258	0.209
APRI	1.53(0.94-2.28)	1.36(0.74-2.77)	0.285	0.776
AAR	1.41(1.06-2.00)	1.17(0.90-1.50)	1.729	0.084
NLR	1.87(1.43-3.23)	2.23(1.83-3.56)	1.120	0.263
PLR	103.87(66.823-123.93)	77.33(60.23-102.38)	1.695	0.090
GPR	1.83(0.53-1.87)	1.09(0.53-1.87)	2.191	0.028
ALT(IU/L)	35.00(19.75-61.25)	28.00(21.50-36.00)	1.322	0.186
AST(IU/L)	56.00(34.50-75.00)	35.00(25.00-49.00)	2.905	0.004
GGT(IU/L)	87.00(37.50-185.25)	30.00(21.25-67.50)	3.346	0.001
ALP(IU/L)	124.00(93.50-229.25)	90.00(70.50-126.00)	3.031	0.002
Total bilirubin(μmol/L)	19.00(14.00-33.25)	20.00(14.00-30.00)	0.118	0.906

AILDs, Autoimmune liver diseases; CHB, Chronic hepatitis B; BMI, Body Mass Index; CHI3L1, Chitinase 3-like protein 1; FIB-4, Fibrosis index based on four factors; APRI, Aspartate aminotransferase-to-platelet ratio index; AAR, Aspartate aminotransferase-to-alanine aminotransferase ratio; NLR, Neutrophil-to-lymphocyte ratio; PLR, Platelet-to-lymphocyte ratio; GPR, Gamma glutamyl transferase-to-platelet ratio; ALT, Alanine aminotransferase; AST, Aspartate aminotransferase; GGT, Gamma glutamyl transferase; ALP, Alkaline phosphatase.

## 4 Discussion

The early detection and continuous monitoring of liver fibrosis play a crucial role in the clinical management and prognostic evaluation of chronic liver diseases. The results of this study indicate that serum CHI3L1 levels are significantly higher in patients with AILDs associated with significant liver fibrosis than in those without significant liver fibrosis. Moreover, there is a strong correlation between serum CHI3L1 levels and LSM in patients with AILDs. When diagnosing significant liver fibrosis, the diagnostic efficacy of serum CHI3L1 is superior to that of other non-invasive diagnostic methods, including APRI, FIB-4, GPR, AAR, NLP, and PLR. It exhibits high specificity and sensitivity, thereby establishing its potential as a reliable biomarker for the accurate diagnosis of fibrosis. Further studies have shown that among patients with liver fibrosis, serum CHI3L1 levels are higher in patients with AILDs than in those with CHB.

In recent years, there has been a steady increase in the annual incidence and mortality rates of AILDs, leading to a significant social and economic burden on patients and their families (21, 22). Routine follow-up assessments for AILDs patients are crucial for guiding management and treatment. Currently, the diagnosis of AILDs relies on liver biopsy, which is not suitable for subsequent follow-up and disease monitoring.

CHI3L1, as a potential biomarker for the diagnosis of liver fibrosis, is implicated in the pathogenesis of hepatic fibrosis. Studies have indicated that fibrosis of various etiologies generally begins with the injury of hepatocytes or cholangiocytes, and the progression of fibrosis is mainly driven by a dysregulated inflammatory process (23). Chronic liver injury leads to persistent inflammation, cellular proliferation, and extracellular matrix protein deposition, ultimately culminating in cirrhosis if left untreated (24, 25). Among these, hepatic macrophages play a central role in liver inflammation and fibrosis (26). During the progression of liver fibrosis, apoptosis of hepatic macrophages is a common response of the body to toxic injury. The death receptor Fas and downstream signal transduction are considered representative extrinsic pathways for macrophage apoptosis (27, 28). In contrast, the persistently active Akt signaling in macrophages is regarded as a primary anti-apoptotic pathway (29). Chitinase 3-like protein 1 is an indicator associated with liver fibrosis in patients with chronic liver diseases research, but its application in the study of AILDs is still not clear (30). It is currently believed that macrophages are the primary source of CHI3L1 (8, 31, 32). Some studies suggest that CHI3L1 inhibits hepatic macrophage apoptosis by suppressing Fas expression and activating the PI3K/Akt signaling pathway, leading to the accumulation and activation of liver macrophages and exacerbating liver fibrosis (8, 33). Additionally, an imbalance in the production and degradation of the extracellular matrix (ECM) results in the abnormal deposition of interstitial collagen and other matrix components in the liver parenchyma, which is a characteristic manifestation of fibrosis (23). CHI3L1 has been shown to inhibit the degradation of collagen and hyaluronic acid, thus promoting ECM accumulation (34–37). This elucidates why serum levels of CHI3L1 are significantly elevated in patients with autoimmune liver disease-related fibrosis compared to healthy controls, and why higher levels are associated with increased severity of fibrosis. However, research on AILDs and CHI3L1 remains limited. Further exploration into molecular and pathological mechanisms is crucial for determining whether CHI3L1 could potentially serve as a target for new fibrosis therapies.

Furthermore, this study also revealed that patients with AILDs and CHB, both exhibiting similar degree of liver fibrosis, often demonstrate elevated serum CHI3L1 levels. This observation may be attributed to the heightened inflammatory activity commonly observed in AILDs patients. These findings suggest that while serum CHI3L1 can serve as a valuable indicator for assessing liver fibrosis in AILDs within clinical settings, it is important to avoid applying a universal diagnostic threshold, particularly when patients exhibit active inflammation, as CHI3L1 values tend to be higher in such cases.

Our study has several limitations. Firstly, due to the retrospective nature of this study and the relatively low incidence of AILDs, the number of patients included was limited, and longitudinal data were lacking. Although we conducted follow - up observations for some patients, the relatively short follow - up duration meant that many patients had not yet reached the clinical endpoints. As a result, the absence of longitudinal data prevented a thorough validation of CHI3L1’s prognostic value in fibrosis monitoring. In addition, since most of the patients included in this study were treated at a single tertiary liver disease center, selection bias might have been introduced. Furthermore, although LSM has a high diagnostic value for liver fibrosis, it may still be affected by factors such as liver congestion, cholestasis, and elevated transaminases (38). These influencing factors also apply to CHI3L1 and might have had a certain impact on our results. Therefore, future research should conduct multic - enter prospective studies to further expand the sample size, verify the diagnostic efficacy of CHI3L1 in AILDs - related liver fibrosis, and determine the diagnostic thresholds for each fibrosis stage. It is also necessary to clarify the factors that affect CHI3L1 when diagnosing and assessing the degree of liver fibrosis. However, in future studies, the control group should be expanded beyond CHB to include alcoholic liver disease and metabolic dysfunction-associated steatotic liver disease, in order to further explore the applicability.

In conclusion, our study revealed a significant elevation in serum CHI3L1 concentration among patients with AILDs-related liver fibrosis compared to those without fibrosis. Furthermore, we observed a positive correlation between serum CHI3L1 levels and the degree of liver stiffness, suggesting a potential association between serum CHI3L1 and liver fibrosis in AILDs patients. We believe that our findings still provide valuable insights into the diagnostic utility of the biomarker in non - tertiary care centers where LSM may not be readily available. Additionally, our findings indicated higher levels of serum CHI3L1 in the AILDs patient group compared to the CHB patient group. These results emphasize the importance of considering inflammatory activity when utilizing serum CHI3L1 as a diagnostic marker in clinical practice, rather than relying on a universal threshold for assessment.

## Data Availability

The original contributions presented in the study are included in the article/supplementary material. Further inquiries can be directed to the corresponding author/s.

## References

[1] Trivedi PJ Hirschfield GM. Recent advances in clinical practice: epidemiology of autoimmune liver diseases. Gut. (2021) 70:1989–2003. doi: 10.1136/gutjnl-2020-322362 34266966

[2] Bonder A Retana A Winston DM Leung J Kaplan MM. Prevalence of primary biliary cirrhosis-autoimmune hepatitis overlap syndrome. Clin gastroenterology hepatology: Off Clin Pract J Am Gastroenterological Association. (2011) 9:609–12. doi: 10.1016/j.cgh.2011.03.019 21440668

[3] Lamba M Ngu JH Stedman CAM. Trends in incidence of autoimmune liver diseases and increasing incidence of autoimmune hepatitis. Clin gastroenterology hepatology: Off Clin Pract J Am Gastroenterological Association. (2021) 19:573–9.e1. doi: 10.1016/j.cgh.2020.05.061 32526342

[4] McGill DB Rakela J Zinsmeister AR Ott BJ. A 21-year experience with major hemorrhage after percutaneous liver biopsy. Gastroenterology. (1990) 99:1396–400. doi: 10.1016/0016-5085(90)91167-5 2101588

[5] Sripongpun P Pongpaibul A Charatcharoenwitthaya P. Value and risk of percutaneous liver biopsy in patients with cirrhosis and clinical suspicion of autoimmune hepatitis. BMJ Open gastroenterology. (2021) 8:e000701. doi: 10.1136/bmjgast-2021-000701 PMC8351491 34362759

[6] EASL Clinical Practice Guidelines on non-invasive tests for evaluation of liver disease severity and prognosis - 2021 update. J hepatology. (2021) 75(3):659–89. doi: 10.1016/j.jhep.2021.05.025 34166721

[7] Liu L Cao J Zhong Z Guo Z Jiang Y Bai Y. Noninvasive indicators predict advanced liver fibrosis in autoimmune hepatitis patients. J Clin Lab analysis. (2019) 33:e22922. doi: 10.1002/jcla.22922 PMC6757115 31115929

[8] Higashiyama M Tomita K Sugihara N Nakashima H Furuhashi H Nishikawa M. Chitinase 3-like 1 deficiency ameliorates liver fibrosis by promoting hepatic macrophage apoptosis. Hepatology research: Off J Japan Soc Hepatology. (2019) 49:1316–28. doi: 10.1111/hepr.v49.11 PMC6916176 31250532

[9] Kjaergaard AD Johansen JS Bojesen SE Nordestgaard BG. Role of inflammatory marker YKL-40 in the diagnosis, prognosis and cause of cardiovascular and liver diseases. Crit Rev Clin Lab Sci. (2016) 53:396–408. doi: 10.1080/10408363.2016.1190683 27187575

[10] Yan L Deng Y Zhou J Zhao H Wang G. Serum YKL-40 as a biomarker for liver fibrosis in chronic hepatitis B patients with normal and mildly elevated ALT. Infection. (2018) 46:385–93. doi: 10.1007/s15010-018-1136-2 PMC5976691 29600444

[11] Wang L Liu T Zhou J You H Jia J. Changes in serum chitinase 3-like 1 levels correlate with changes in liver fibrosis measured by two established quantitative methods in chronic hepatitis B patients following antiviral therapy. Hepatology research: Off J Japan Soc Hepatology. (2018) 48:E283–e90. doi: 10.1111/hepr.12982 28895260

[12] Jin X Fu B Wu ZJ Zheng XQ Hu JH Jin LF. Serum chitinase-3-like protein 1 is a biomarker of liver fibrosis in patients with chronic hepatitis B in China. Hepatobiliary pancreatic Dis international: HBPD Int. (2020) 19:384–9. doi: 10.1016/j.hbpd.2020.05.009 32540209

[13] Hennes EM Zeniya M Czaja AJ Parés A Dalekos GN Krawitt EL. Simplified criteria for the diagnosis of autoimmune hepatitis. Hepatology (Baltimore Md). (2008) 48:169–76. doi: 10.1002/hep.22322 18537184

[14] European Association for the Study of the Liver. EASL Clinical Practice Guidelines: The diagnosis and management of patients with primary biliary cholangitis. J hepatology. (2017) 67(1):145–72. doi: 10.1016/j.jhep.2017.03.022 28427765

[15] Chazouillères O Wendum D Serfaty L Montembault S Rosmorduc O Poupon R. Primary biliary cirrhosis-autoimmune hepatitis overlap syndrome: clinical features and response to therapy. Hepatology (Baltimore Md). (1998) 28:296–301. doi: 10.1002/hep.510280203 9695990

[16] Sarin SK Kumar M Lau GK Abbas Z Chan HL Chen CJ. Asian-Pacific clinical practice guidelines on the management of hepatitis B: a 2015 update. Hepatology Int. (2016) 10:1–98. doi: 10.1007/s12072-015-9675-4 PMC4722087 26563120

[17] Rinella ME Lazarus JV Ratziu V Francque SM Sanyal AJ Kanwal F. A multisociety Delphi consensus statement on new fatty liver disease nomenclature. J hepatology. (2023) 79:1542–56. doi: 10.1016/j.jhep.2023.06.003 37364790

[18] Zou H Ma X Pan W Xie Y. Comparing similarities and differences between NAFLD, MAFLD, and MASLD in the general U.S. population. Front Nutr. (2024) 11:1411802. doi: 10.3389/fnut.2024.1411802 39040926 PMC11260733

[19] Huang LL Yu XP Li JL Lin HM Kang NL Jiang JJ. Effect of liver inflammation on accuracy of FibroScan device in assessing liver fibrosis stage in patients with chronic hepatitis B virus infection. World J gastroenterology. (2021) 27:641–53. doi: 10.3748/wjg.v27.i7.641 PMC7901051 33642834

[20] Eddowes PJ Sasso M Allison M Tsochatzis E Anstee QM Sheridan D. Accuracy of fibroScan controlled attenuation parameter and liver stiffness measurement in assessing steatosis and fibrosis in patients with nonalcoholic fatty liver disease. Gastroenterology. (2019) 156:1717–30. doi: 10.1053/j.gastro.2019.01.042 30689971

[21] Carbone M Neuberger JM. Autoimmune liver disease, autoimmunity and liver transplantation. J hepatology. (2014) 60:210–23. doi: 10.1016/j.jhep.2013.09.020 24084655

[22] Sharma R Verna EC Söderling J Roelstraete B Hagström H Ludvigsson JF. Increased mortality risk in autoimmune hepatitis: A nationwide population-based cohort study with histopathology. Clin gastroenterology hepatology: Off Clin Pract J Am Gastroenterological Association. (2021) 19:2636–47.e13. doi: 10.1016/j.cgh.2020.10.006 PMC9347643 33065308

[23] Matsuda M Seki E. The liver fibrosis niche: Novel insights into the interplay between fibrosis-composing mesenchymal cells, immune cells, endothelial cells, and extracellular matrix. Food Chem toxicology: an Int J published Br Industrial Biol Res Association. (2020) 143:111556. doi: 10.1016/j.fct.2020.111556 PMC7484466 32640349

[24] Zhang D Zhang Y Sun B. The molecular mechanisms of liver fibrosis and its potential therapy in application. Int J Mol Sci. (2022) 23(20):12572. doi: 10.3390/ijms232012572 PMC9604031 36293428

[25] Wang Z Du K Jin N Tang B Zhang W. Macrophage in liver Fibrosis: Identities and mechanisms. Int immunopharmacology. (2023) 120:110357. doi: 10.1016/j.intimp.2023.110357 37224653

[26] Jangra A Kothari A Sarma P Medhi B Omar BJ Kaushal K. Recent advancements in antifibrotic therapies for regression of liver fibrosis. Cells. (2022) 11(9):1500. doi: 10.3390/cells11091500 PMC9104939 35563807

[27] Fox CK Furtwaengler A Nepomuceno RR Martinez OM Krams SM. Apoptotic pathways in primary biliary cirrhosis and autoimmune hepatitis. Liver. (2001) 21:272–9. doi: 10.1034/j.1600-0676.2001.021004272.x 11454191

[28] Tsikrikoni A Kyriakou DS Rigopoulou EI Alexandrakis MG Zachou K Passam F. Markers of cell activation and apoptosis in bone marrow mononuclear cells of patients with autoimmune hepatitis type 1 and primary biliary cirrhosis. J hepatology. (2005) 42:393–9. doi: 10.1016/j.jhep.2004.11.023 15710223

[29] Liu H Perlman H Pagliari LJ Pope RM. Constitutively activated Akt-1 is vital for the survival of human monocyte-differentiated macrophages. Role of Mcl-1, independent of nuclear factor (NF)-kappaB, Bad, or caspase activation. J Exp medicine. (2001) 194:113–26. doi: 10.1084/jem.194.2.113 PMC2193455 11457886

[30] Johansen JS Christoffersen P Møller S Price PA Henriksen JH Garbarsch C. Serum YKL-40 is increased in patients with hepatic fibrosis. J hepatology. (2000) 32:911–20. doi: 10.1016/S0168-8278(00)80095-1 10898311

[31] Montgomery TA Xu L Mason S Chinnadurai A Lee CG Elias JA. Breast Regression Protein-39/Chitinase 3-Like 1 Promotes Renal Fibrosis after Kidney Injury via Activation of Myofibroblasts. J Am Soc Nephrology: JASN. (2017) 28:3218–26. doi: 10.1681/ASN.2017010110 PMC5661290 28679671

[32] Kawada M Seno H Kanda K Nakanishi Y Akitake R Komekado H. Chitinase 3-like 1 promotes macrophage recruitment and angiogenesis in colorectal cancer. Oncogene. (2012) 31:3111–23. doi: 10.1038/onc.2011.498 PMC3290745 22056877

[33] Zhao H Huang M Jiang L. Potential roles and future perspectives of chitinase 3-like 1 in macrophage polarization and the development of diseases. Int J Mol Sci. (2023) 24:16149. doi: 10.3390/ijms242216149 PMC10671302 38003338

[34] Eurich K Segawa M Toei-Shimizu S Mizoguchi E. Potential role of chitinase 3-like-1 in inflammation-associated carcinogenic changes of epithelial cells. World J gastroenterology. (2009) 15:5249–59. doi: 10.3748/wjg.15.5249 PMC2776850 19908331

[35] Prakash M Bodas M Prakash D Nawani N Khetmalas M Mandal A. Diverse pathological implications of YKL-40: answers may lie in ‘outside-in’ signaling. Cell signalling. (2013) 25:1567–73. doi: 10.1016/j.cellsig.2013.03.016 23562456

[36] Zhao T Su Z Li Y Zhang X You Q. Chitinase-3 like-protein-1 function and its role in diseases. Signal transduction targeted Ther. (2020) 5:201. doi: 10.1038/s41392-020-00303-7 PMC7490424 32929074

[37] Iwata T Kuwajima M Sukeno A Ishimaru N Hayashi Y Wabitsch M. YKL-40 secreted from adipose tissue inhibits degradation of type I collagen. Biochem Biophys Res communications. (2009) 388:511–6. doi: 10.1016/j.bbrc.2009.08.024 19666003

[38] European Association for Study of Liver; Asociacion Latinoamericana para el Estudio del Higado. EASL-ALEH Clinical Practice Guidelines: Non-invasive tests for evaluation of liver disease severity and prognosis. J hepatology. (2015) 63(1):237–64. doi: 10.1016/j.jhep.2015.04.006 25911335

